# Microfluidic devices for on-chip quantification of extracellular vesicles and associated biomarkers

**DOI:** 10.20517/evcna.2025.10

**Published:** 2025-09-12

**Authors:** Víctor Calero, Carlos Honrado, Alar Ainla, Daniela Macedo, Hugo Silva, Teresa Lage, Sara Abalde-Cela, Lorena Diéguez, María Carmen Blanco-López, Esther Serrano-Pertierra

**Affiliations:** ^1^Department of Applied Physics and Institute of Biotechnology of Asturias, University of Oviedo, Oviedo 33006, Spain.; ^2^Medical Devices Research Group, International Iberian Nanotechnology Laboratory, Braga 4715-330, Portugal.; ^3^Department of Physical and Analytical Chemistry and Institute of Biotechnology of Asturias, University of Oviedo, Oviedo 33006, Spain.; ^4^Department of Biochemistry and Molecular Biology and Institute of Biotechnology of Asturias, University of Oviedo, Oviedo 33006, Spain.; ^#^Authors contributed equally.

**Keywords:** Extracellular vesicles, microfluidics, on-chip quantification, molecular biomarkers, lab-on-a-chip, integrated sensing

## Abstract

In the past decade, extracellular vesicles (EVs) have gained increasing attention in biomedical research. These membrane-bound particles are naturally secreted by cells under both physiological and pathological conditions, and they exhibit a wide range of sizes and molecular compositions. EVs transport bioactive molecules - such as proteins, nucleic acids, and lipids - making them ideal candidates for biomarker discovery. Consequently, their accurate characterization and quantification are critical for understanding their roles in intercellular communication and evaluating their potential in diagnostics, prognostics, disease monitoring, and therapeutic applications. Microfluidic technologies offer promising solutions for EV analysis, addressing key limitations of conventional methods by enabling precise and sensitive measurements with small sample volumes. While microfluidic devices have been predominantly used for EV separation and isolation, their application in EV quantification remains underexplored. Compared to traditional techniques like nanoparticle tracking analysis or flow cytometry, microfluidic systems can provide faster, more accessible alternatives for EV quantification. This review summarizes recent advances in microfluidic technologies for EV quantification, discussing their advantages, current limitations, and future prospects.

## INTRODUCTION

**Figure 1:**
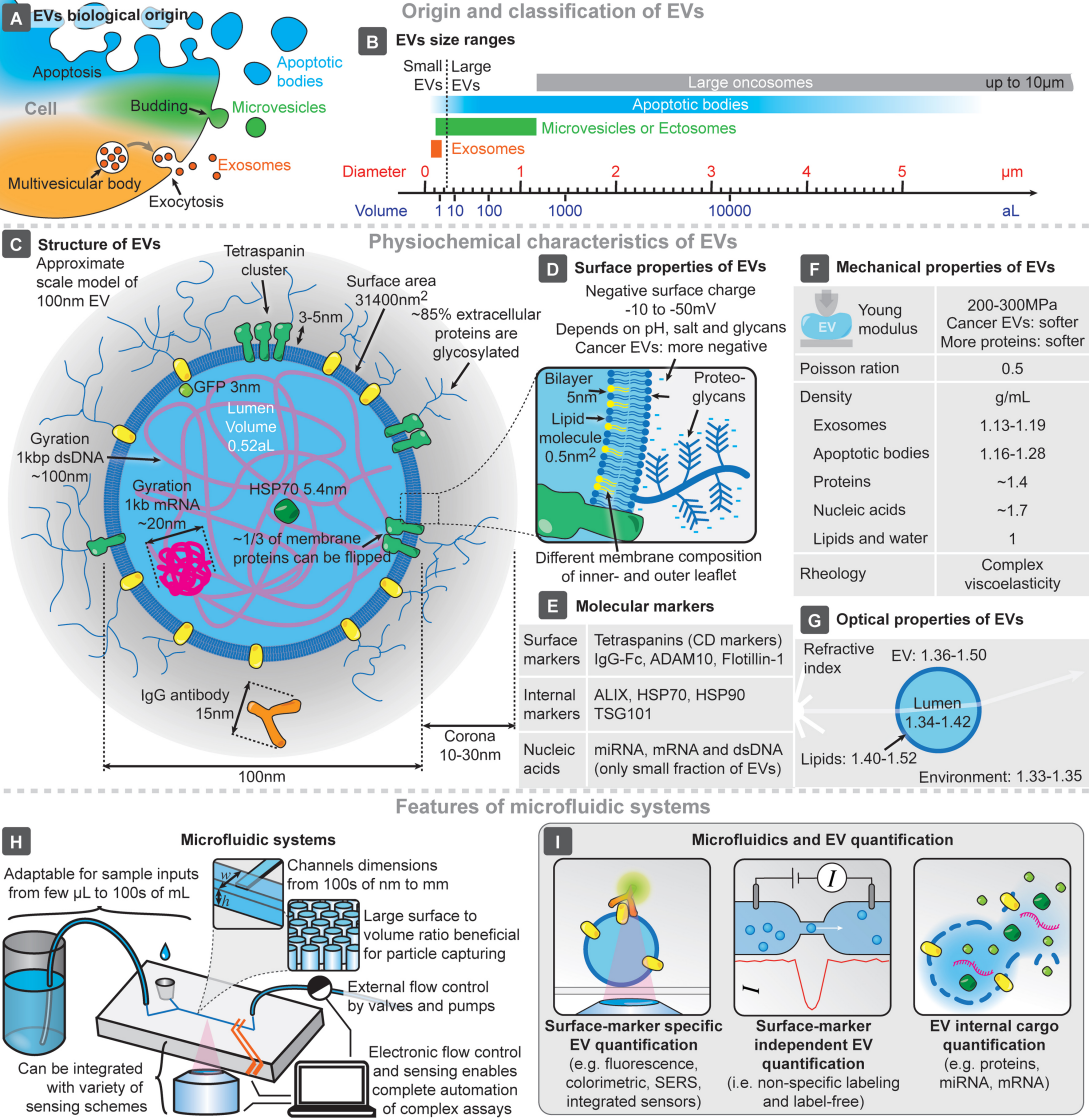
Main features and characteristics of EVs and microfluidic systems. Origin (A) and size range (B) of EVs. (C) Representative structure of a 100 nm EV, compared to the sizes of its typical molecular components. Main surface (D), molecular (E), mechanical (F), and optical (G) characteristics of EVs. Key features of microfluidic systems and chips (H), and highlighted approaches for microfluidic EV quantification (I). EVs: Extracellular vesicles; SERS: surface-enhanced raman spectroscopy.

Intracellular compartmentalization of chemical processes within membrane-enclosed reaction vessels is essential to eukaryotic cells, and nearly all cells release small vesicles into their surroundings. Although extracellular vesicles (EVs) have been known for over half a century^[1-3]^, recent research has revealed their crucial roles in intercellular communication, particularly their therapeutic and diagnostic potential^[4]^. Despite the potential benefits of using EVs as biomarkers, their clinical application faces challenges in standardization and reproducibility of isolation, detection, and quantification, largely due to their small size (typically 50-1,000 nm), abundance, and physicochemical properties^[5]^. EVs are typically classified based on their origin [Figure 1A] or size [Figure 1B]^[6]^, given the preponderance of size in isolation techniques (e.g., centrifugation and chromatography-based approaches). Their structure is known to replicate many (but possibly not all) of the features of their cells of origin^[7]^, carrying with them various biomarkers, both internally and at the surface [Figure 1C-E]. Of note, the concentration of tetraspanins in EV membranes can exceed that of their cell of origin^[8]^, while lipidic composition is also distinct from cells, with a dense proteoglycan shell surrounding the membrane^[9]^, with these characteristics being thus explored for both EV isolation and quantification. EVs also have distinct mechanical properties [Figure 1F], essential for gold-standard isolation methods based on centrifugation, and optical properties [Figure 1G], which are exploited by typical technologies for EV quantification and characterization (e.g., nanoparticle tracking analysis - NTA)^[6,10]^.

However, the standardization of technologies and procedures to ensure comparable and reproducible results remains one of the major challenges in EV-related research. To address this, the International Society of Extracellular Vesicles (ISEV) provides recommendations and guidelines in this field^[6]^, not only specifying the minimum EV sample characterization required, but also advocating for consistent nomenclature. Regarding the latter, the use of the term EV is recommended when specific markers of biogenesis pathways are not identified. Researchers are thus encouraged to describe EVs according to other parameters, such as size, biochemical composition, or characteristics related to their cells of origin. In this review, the terms “exosome” and “EV” were used in accordance with the terminology employed by the authors of the works discussed herein.

The widespread use of current “gold-standard” methods for EV quantification, such as NTA, results in well-established protocol guidelines^[6]^. NTA is typically used to characterize particle size and concentration. However, in complex matrices like human samples (i.e., blood, plasma, serum), the presence of non-vesicular extracellular particles significantly complicates NTA analyses. Consequently, NTA is often unsuitable for clinical applications due to its cumbersome use and high upfront cost^[5]^. In contrast, while microfluidic platforms are in an earlier stage of development and implementation than NTA, they offer greater flexibility in the analytical information that can be obtained from EVs, including cargo and protein analysis, nucleic acids, and complex omic studies.

This advantage drives the development of seamless EV quantification technologies. Microfluidics enables the integration, miniaturization, and automation of complex biochemical analyses, leveraging the small feature sizes of microfluidic systems (from 100s of nm to a few mm - Figure 1H)^[11,12]^. The design and fabrication flexibility of microfluidics allows for the development of systems that manipulate microscopic objects while reducing material costs. Devices can be designed with large surface-to-volume ratios, enhancing EV absorption, and can be tailored for specific EV capture schemes^[13,14]^. Furthermore, the integration of external/internal sensing approaches enables the analysis of individual captured particles. This flexibility also allows for high-throughput processing of both small and large volumes of complex samples, a critical consideration for clinical applications. Thus, the strong synergy between EVs and microfluidics is evident. At the intersection of these two fields, a major focus has been on the isolation of EVs, exploring how phenomena at the microscale could enhance this process^[13-16]^. Moreover, applying microfluidic devices for the analysis and characterization of EVs and their cargo/biomarkers has become a key area of interest in recent years^[13-18]^. Other EV-related efforts have been utilized in a variety of fields, including regenerative medicine^[19]^ or liquid biopsy and oncology^[20-22]^, for example. Within these broader trends, there is an increasing interest in not only performing the on-chip isolation of EVs, but also in integrating the capability to perform on-chip quantification, analysis, and characterization of isolated EVs. This review provides a concise summary of recent advances and current trends in microfluidics-based EV quantification technologies, covering both surface marker-specific and -independent approaches, as well as the quantification of EV internal cargo - Figure 1I.

## SURFACE MARKER-SPECIFIC EV QUANTIFICATION

EV surface proteins can be targeted for quantification, characterization, functional analysis, and biomarker studies. Transmembrane proteins, such as the commonly found tetraspanins CD63, CD9, and CD81, are often used, and pathology-specific markers can also be targeted for EV quantification. The main features of surface marker-specific quantification platforms are summarized in Table 1.

**Table 1:** Summary of techniques developed for microfluidic on-chip quantification based on surface markers

**Authors**	**Type**	**Principle**	**Performance parameters**	**Sample type**	**Sample pre-processing**	**Markers targeted**
Chen *et al*.^[28]^	Fluorescence	Membrane EV isolation/counting microfluidic platform	LOD: 10^5^ particles/mL (10^8^ for clinical samples) Range: 1 × 10^5^-4 × 10^6^ particles/mL Volume: 2 µL	Whole blood	N	CD63
Chen *et al*.^[27]^	Fluorescence	Membrane-based filtration + magnetic-bead based immunoassay	LOD: 3 × 10^10^ particles/mL Linear range: 1-6 × 10^12^ particles/mL	Whole blood	N	CD63
Yang *et al*.^[24]^	Fluorescence	Droplet-based optofluidic platform	LOD: 9 × 10^3^ particles/mL	FBS spiked with neuronal EVs	N	CD81
Liu *et al*.^[29]^	Fluorescence	Droplet-based single-exosome counting immunoassay	LOD: 10^4^ particles/mL Range: 10^4^-10^8^ particles/mL	Human serum	N	CD63 to capture and GPC-1 to detect
Wu *et al*.^[31]^	Fluorescence	Fluorescence immunoassay on a herringbone structure	LOD (based on protein concentration): 9.95 ng/µL	Mice plasma	N	CD63 and PD-L1
Walker *et al*.^[30]^	Fluorescence	Fluorescently labeled EVs captured in a NPN membrane	Analyzes up to 10^4^ EVs Selective detection of tumor-related Evs - 1%	Human plasma CM	Labeled and prefiltered	CD9 and ICAM-1
Ji *et al*.^[33]^	Fluorescence	Magnetic EV isolation, Acoustic wave-assisted mixing; Monolayer-fluorescence counting using a step-wedge	LOD: 8.5 × 10^2^ particles/mL	CM Saliva	N	EpCAM
Zhao *et al*.^[23]^	Fluorescence	DEP trapping in microwells	LOD: 193 particles/mL Linear range: 1.4 × 10^3^-1.4 × 10^8^ particles /mL	CM	UC	CD63 to trap and to detect: CD81, CEA, EpCAM, CD147, and AFP
Lee *et al*.^[25]^	Fluorescence	Single EV analysis with multiplexed immunofluorescence	Simultaneous analysis of > 10^3^ EV Detection of 11 markers	CM	Differential centrifugation, filtration	CD9, CD63, CD81, EGFR, EGFRvIII, IDH1, IDH1R132, PDPN, PDGFRα, PD-L1, PD-L2
Zhou *et al*.^[32]^	Chemiluminescence	Inertial separation + capture on AuNPs + chemiluminescence	LOD: 9.5 × 10^4^ particles/mL Range: 2.5 × 10^5^-2.5 × 10^11^ particles/mL	Whole blood	N	CD24 CD81, EpCAM
Wang *et al*.^[34]^	Colorimetric	Centrifugal chip + capture on magnetic NPs	LOD: 10^6^ particles/mL Volume: 100 μL of blood Time: 10 min	CM Plasma (human) Blood (mice)	UC	CD63 to capture CEA, CA125, EGFR
Vaidyanathan *et al*.^[35]^	Colorimetric	Capture with AC-EHDs + Catalytic oxidation of HRP with TMB	LOD: 2.8 × 10^6^ particles/mL	CM	Exosome isolation reagent	CD81 and HER2
Stollmann *et al*.^[36]^	Digital in-line holography	Optofluidics	Range: 1.9 × 10^8^ to 7.6 × 10^9^ particles/mL Throughput: 10^4^ events/h	CM	Ultrafiltration and SEC	CD63, CD9, CD81, EpCAM, CA-125, HE-4
Cavallaro *et al*.^[38]^	Electrical	Electrical current in a capillary	LOD: 2.8 × 10^8^ particles/mL	CM	SEC or UC	CD63, CD9, EGFR
Kim *et al*.^[40]^	Electrical	Inertial and DEP separation + GO biosensor	Linear range: 10^4^ to 10^6^ particles/mL	CM	N	CD63
Talebian Gevari *et al*.^[39]^	Electrical	Streaming current biosensing	LOD: 10^4^ particles/mL Size range 30-200 nm	CM Plasma	SEC	CD9, CD81, CD73, and PD-L1
Li *et al*.^[42]^	Electrochemical	Electrochemical microfluidic aptasensor	LOD: 1.4 × 10^4^ particles/mL Linear range: 10^5^-10^9^ particles/mL Time: 60 min	CM Plasma	Exosome extraction Kit	EpCAM aptamer
Wang *et al*.^[41]^	Electrochemical	Dual microfluidic filtration chip + four SPEs	LOD: 1 × 10^4^ particles/mL Time: 60 min	Whole blood	N	PMSA, EGFR, CD81, and CEA
Qian *et al*.^[43]^	Magnetic	DNA-mediated magnetic detection	LOD: 1.98 × 10^3^ particles/mL Linear Range: 10^3^-10^7^ particles/mL Detection time: ~1 h	CM Simulated serum	UC and filtration	CD9, CD63
Ho *et al*.^[46]^	SERS	Droplet microfluidics + SERS	LOD: 10^4.5^ particles/mL Time: 5 min	CM Plasma	N	HER2, CD44, CD63
Wang *et al*.^[44]^	SERS	SERS microfluidic biosensor	LOD (in particles/mL): MCSP/ MCAM - 1 × 10^3^, CD61/CD63 - 1 × 10^5^ Volume: 5 µL	CM Plasma	CM - SEC + UF Plasma - tenfold dilution	MCSP, MCAM, CD61, CD63
Zhou *et al*.^[45]^	SERS	SERS microfluidic biosensor	LOD: 10^6^ particles/mL for LacdiNAc and T antigen and 10^5^ particles/mL for CD81	CM Plasma	SEC and UC	CD81, LacdiNAc, T antigen
Hao *et al*.^[47]^	SERS	Acoustic enrichment + immunofluorescent/SERS bimodal biosensors	Fluorescence mode - LOD: 1.3 × 10^6^ particles/mL SERS mode - LOD: 2 × 10^4^ particles/mL Linear range: 10^5^-10^11^ particles/mL	Commercial solution (EV standard) Plasma	Acoustic isolation for plasma samples	CD63

CEA: Carcinoembryonic antigen; DEP: dielectrophoresis; EGFR: epidermal growth factor receptor; EpCAM: epithelial cell adhesion molecule; EVs: Extracellular vesicles; FBS: fetal bovine serum; GO: graphene oxide; MCAM: melanoma cell adhesion molecule; MCSP: melanoma-associated chondroitin sulfate proteoglycan; NPN: nanoporous silicon nitride; NP: nanoparticle; PDPN: podoplanin; PSMA: prostate-specific membrane antigen; SERS: surface-enhanced Raman scattering; SPE: screen-printed electrode; LOD: limit of detection; CM: conditioned media; UC: ultracentrifugation; UF: ultrafiltration; SEC: size exclusion chromatography; AC-EHD: alternating current electrohydrodynamics; AuNPs: gold nanoparticles; HRP: horseradish peroxidase; TMB: 3,3’,5,5’-tetramethylbenzidine.

### Fluorescence

Many microfluidic techniques for surface biomarker-based EV quantification use fluorescent labeling to measure the intensity of captured vesicles. This enables a correlation between biomarker abundance and fluorescence signal intensity. Additionally, when targeting common EV markers (e.g., tetraspanins), these methods provide an estimate of the total EV concentration.

For example, a system using anti-CD63 labeling and samples from A549 cell culture supernatant achieved a limit of detection (LOD) of 193 exosomes/mL, with the relative abundance of other surface proteins (CD81, CD147, EpCAM, CEA, and AFP) also being assessed^[23]^. A droplet-based microfluidic platform was shown to provide information from single CD81^+^ EVs from human induced pluripotent stem cells differentiated to neurons (LOD: 9 × 10^3^ EVs/mL)^[24]^. Other systems have been optimized for single vesicle detection and analysis of multiple surface markers by immunofluorescence using previously isolated EVs from human glioblastoma cell lines^[25]^. Another system simultaneously assessed EV sample purity, particle size, and EV quantities with CD63^+^ EVs isolated from a mesenchymal stem cell line within a range of 1.25-5 × 10^13^ particles/mL^[26]^.

**Figure 2:**
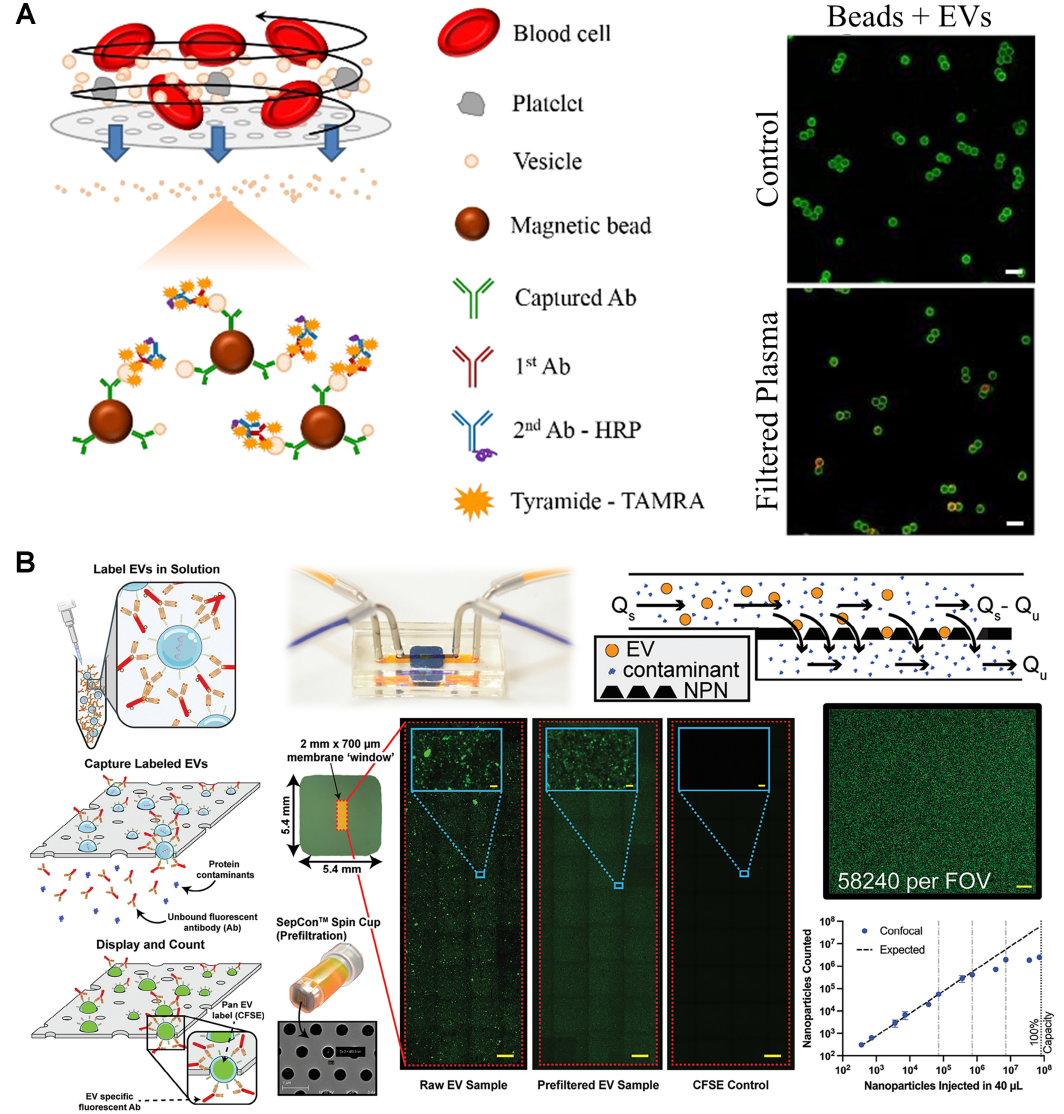
Fluorescence-based approaches for surface marker-based EV quantification. (A) ELISA-based EV quantification from blood samples (adapted from ref.^[27]^); (B) Detection of fluorescent EVs trapped on ultrathin nanopore membrane (adapted from ref.^[30]^). EVs: Extracellular vesicles; ELISA: enzyme-linked immunosorbent assay; HRP: horseradish peroxidase; NPN: nanoporous silicon nitride; FOV: field of view.

Some microfluidic platforms can use whole blood directly by integrating a filtration module to obtain platelet-free plasma. For example, circulating EVs can be captured using an ELISA-based method, with EV quantification based on the fluorescence intensity of CD63-labeled EVs^[27,28]^. With only 2 µL of whole blood, the system achieved a LOD of 10^5^ CD63^+^ EVs/mL^[28]^ - Figure 2A. In a different approach, a droplet microfluidics system used magnetic beads for EV capture, followed by quantification of CD63^+^GPC-1^+^ exosomes from breast cancer patient serum (LOD: 10^4^ exosomes/mL)^[29]^. A microfluidic system that showed good results for counting and characterizing EVs from both cell cultures and plasma samples with minimal handling has also been developed^[30]^ - Figure 2B. Labeled EVs with CFSE were detected in a linear range of 10^2^-10^6^ EVs (from cell cultures) and 10^2^-10^4^ EVs (from plasma). For further characterization, colocalization studies for the surface markers CD9, ICAM-1, PD-1, and PD-L1 were also conducted^[30]^. Another interesting strategy was developed to monitor nascent EVs upon the administration of an immunotherapy^[31]^. The combination of metabolic labeling of EVs and detection of PD-L1^+^ and PD-L1^+^CD63^+^ plasma-derived EVs that were secreted over time in response to an anti-PD-L1 treatment in a mouse model was performed. With the metabolic labeling approach, the chip achieved a LOD of 9.95 ng/µL (quantified by the bicinchoninic acid protein assay). Chemiluminescence was also explored to quantify CD63^+^CD81^+^ EVs (LOD: 9.5 × 10^4^ EVs/mL), as well as the levels of CD24^+^ and EpCAM^+^ EVs in cell lines and in plasma samples from patients with ovarian cancer and healthy controls^[32]^.

This family of techniques has also been extended to other biofluids. Saliva-derived EVs expressing EpCAM were detected in a platform where the sandwich complexes formed between magnetic beads, fluorescent beads, and EpCAM^+^ EVs were arranged in a monolayer and counted individually. This method achieved a LOD of 850 particles/mL and was tested in samples from healthy controls and oral cancer patients^[33]^.

### Colorimetric and holography

**Figure 3:**
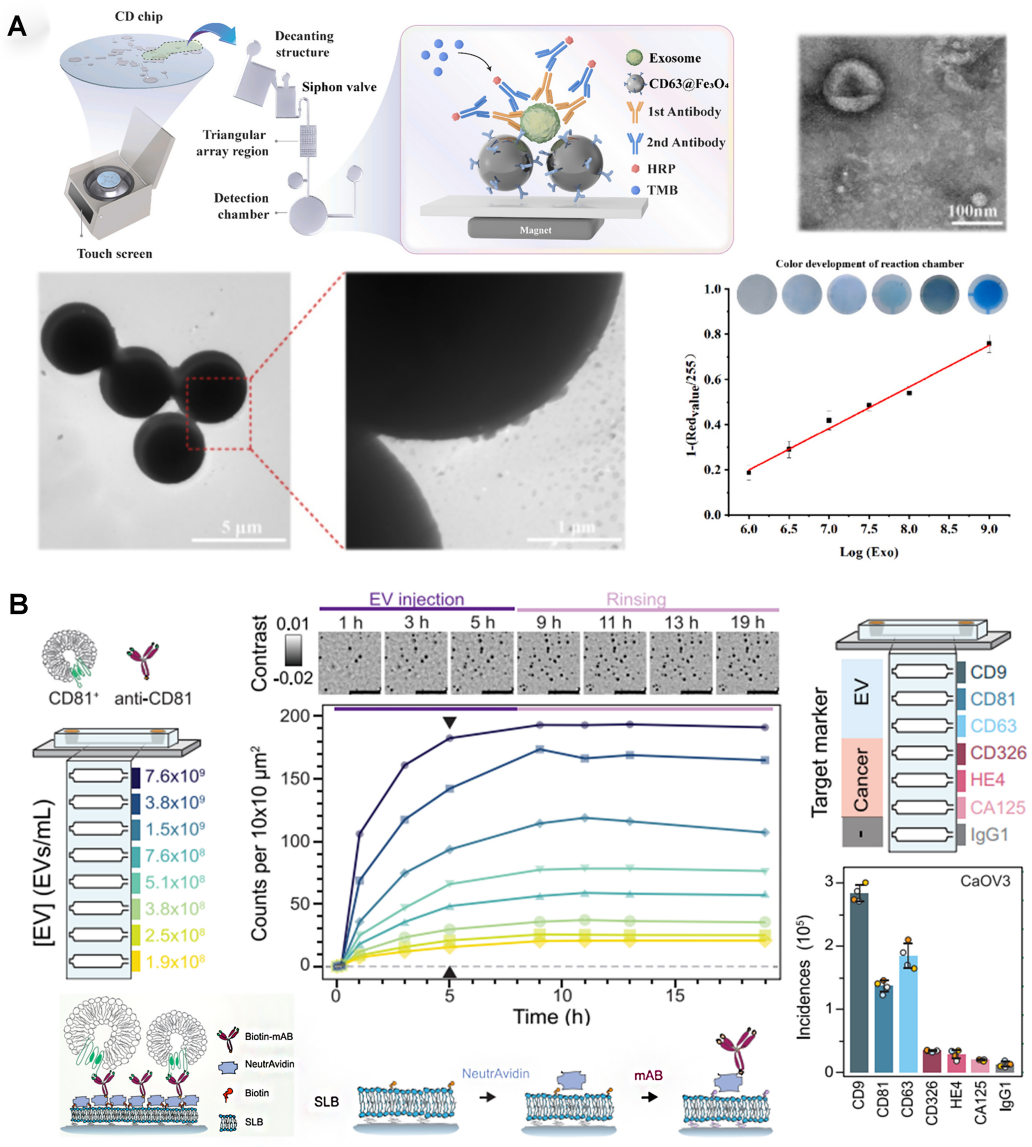
Colorimetric and holography-based approaches for surface marker-based EV quantification. (A) Colorimetric EV quantification in centrifugal microfluidic disk (adapted from ref.^[34]^); (B) Digital in-line holography device for EV detection (adapted from ref.^[36]^). EVs: Extracellular vesicles; HRP: horseradish peroxidase; TMB: 3,3’,5,5’-tetramethylbenzidine.

Instead of fluorescence emission, colorimetric changes can also quantify EVs within a device. A centrifugal disk system capable of efficient EV capturing (using CD63-targeting carboxyl magnetic beads) was integrated with EV labeling (antibodies targeting CEA, CA125, and EGFR) for on-chip detection based on a horseradish peroxidase-associated secondary antibody^[34]^ - Figure 3A. The system handled small volumes (100 μL) of blood samples, providing visual results within 10 min with a LOD of 1 × 10^6^ particles/mL. The Trau group used an electrohydrodynamic device to create fluid flow a few nanometers away from an electrode surface; the induced surface shear forces (“nanoshearing”) improved immuno-based EV capture at the electrode surface^[35]^. Using the catalytic oxidation of horseradish peroxidase, a rapid naked-eye colorimetric assessment of EV capture is possible, as well as UV-visible spectroscopy measurements, for the analysis of multiple EV biomarkers (e.g., HER2 and PSA) with a LOD of 2.8 × 10^6^ EVs/mL.

Digital in-line holography can also be integrated for on-chip EV quantification, providing high-throughput molecular fingerprint - Figure 3B^[36]^. This optofluidic system contained a liposome-covered substrate, treated with a biotin-based protocol for antibody-based EV capture. By targeting EV-specific and cancer-specific biomarkers for four different ovarian cancer subtypes, the system could identify the presence of EVs with concentrations above 1 × 10^8^ particles/mL, creating a unique fingerprint for each cancer subtype.

### Electrochemical and magnetic sensors

Electrochemical and magnetic sensing are alternative strategies for surface marker-specific EV quantification. These methods use affinity-based detection mechanisms to capture and analyze EVs via electrochemical or magnetic signal transduction. Electrochemical techniques offer high-sensitivity detection by measuring changes in electrical properties upon EV binding, while magnetic sensors use nanoparticle-based labeling for enhanced signal amplification. These sensors often provide higher sensitivity than fluorescence methods and can be used with portable readers, eliminating the need for large equipment like the fluorescence microscopes required for most fluorescence-based methods.

**Figure 4:**
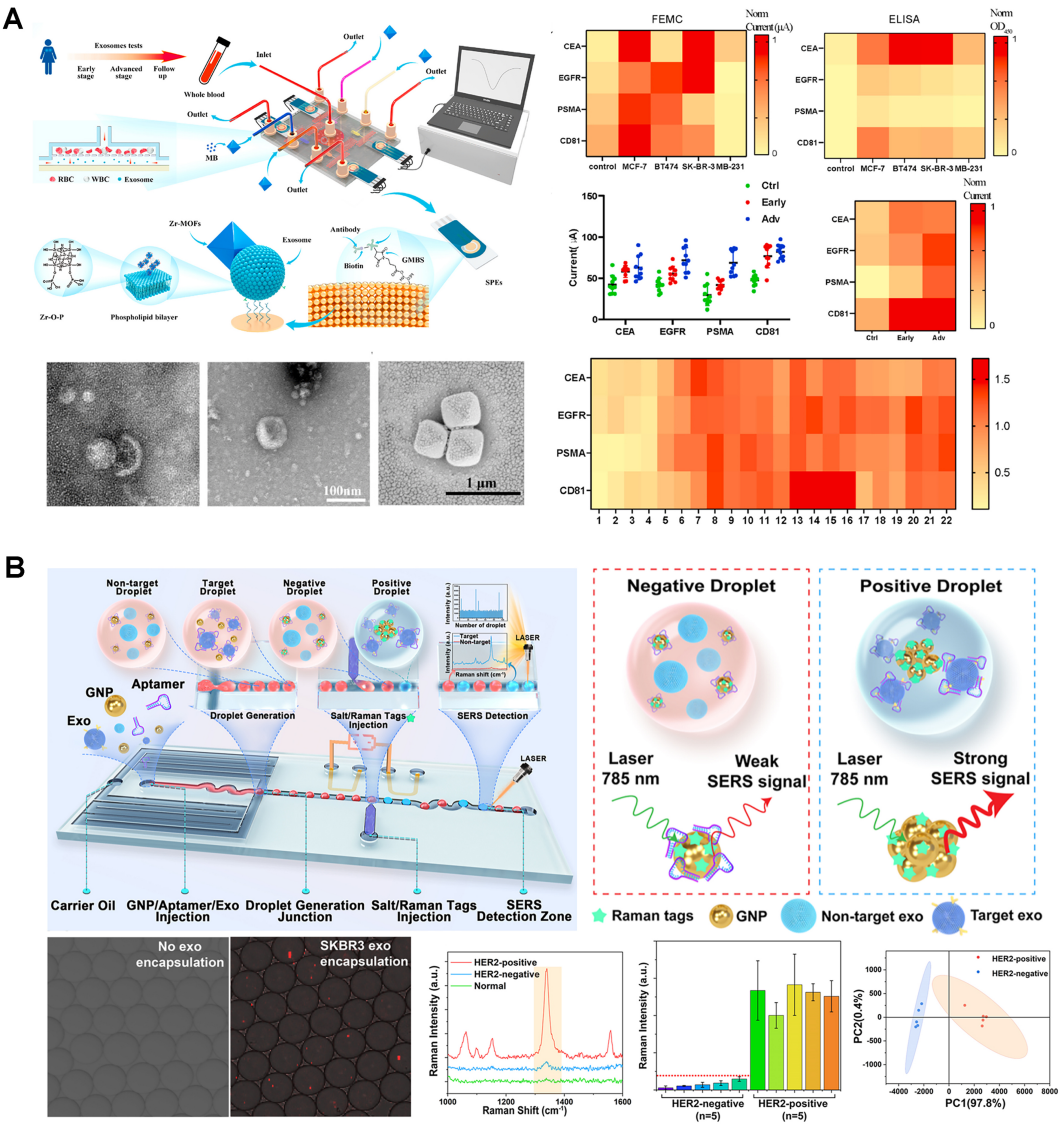
Integrated sensors and SERS approaches for surface marker-based EV quantification. (A) Electrochemical EV quantification (adapted from ref.^[41]^); (B) Droplet microfluidics with integrated SERS for EV detection (adapted from ref.^[46]^). EVs: Extracellular vesicles; SERS: surface-enhanced raman spectroscopy.

Cavallaro *et al*. targeted EGFR and CD63, using an electrokinetic sensor. In this device, changes in the streaming current produced by the affinity-based binding of EVs to the walls of a microcapillary were used for quantification (LOD of 2.8 × 10^8^ particles/mL)^[37]^. This device was subsequently adapted for simultaneous detection of multiple EV surface markers^[38]^. In a follow-up study, it was functionalized with silane-PEG-biotin to detect PD-L1^+^ and CD73^+^ EVs (LOD of 1.4 × 10^4^ particles/mL), including detection in plasma samples from a lung cancer patient^[39]^. Dielectrophoretic (DEP) forces, generated within an Ag-doped microfluidic device, and inertial effects have also been used for EV separation (83% efficiency), followed by EV detection using a CD63-based reduced graphene oxide-based biosensor (linear range of 10^4^ to 10^6^ particles/mL)^[40]^. Another approach employed antibody-coated screen-printed electrodes to capture isolated EVs, followed by the adsorption of metal-organic frameworks onto the EV surface, leading to the amplification of electrical signals^[41]^. The system enabled the detection of EV-associated tumor biomarkers and the classification of various breast cancer mouse models and clinical samples (LOD: 1 × 10^4^ particles/mL) - Figure 4A. An integrated electrochemical microfluidic aptasensor, based on impedance spectroscopy, was shown to identify differences between EVs from lung cancer and healthy controls^[42]^. Using herringbone structures to enhance EpCAM-based EV capture onto the gold electrode, a biomolecular layer was formed after adsorption, causing an increase in impedance proportional to EV concentration (LOD: 1.4 × 10^4^ particles/mL). A microfluidic magnetic detection system was also used to quantify EVs^[43]^. EVs were captured through a sandwich assay using a DNA tetrahedral-structured probe connected to streptavidin and biotin-anti-CD9, followed by labeling using CD63 aptamer-modified Fe_3_O_4_ magnetic nanoparticles. An induction coil-based magnetic detector was then able to measure the EV-associated magnetic signal (LOD: 1.98 × 10^3^ particles/mL).

### Surface-enhanced raman spectroscopy

Recent works have explored the use of surface-enhanced raman spectroscopy (SERS) systems for EV quantification. In one such study, the Trau group employed a nanoshearing device to capture and quantify EVs based on melanoma-associated biomarkers: MCSP and MCAM (LOD: 1 × 10^3^ particles/mL), and CD61 and CD63 (LOD: 1 × 10^5^ particles/mL)^[44]^. Subsequently, SERS signatures of the captured EVs were obtained from a cohort of 20 early-stage melanoma patients and 21 healthy controls. A mean fold increase in SERS signal intensity ranging from 3.7 to 4.2 was observed in patients compared to controls. The nanoshearing system was later adapted for multiplex profiling of glycans in EV membranes from non-small cell lung cancer patients^[45]^. Two different glycans (LacdiNAc and T antigen), plus CD81, were used to label SERS nanotags for EV characterization. SERS signatures from EV glycans were assessed for 40 patients (LOD: 1 × 10^6^ particles/mL) and revealed different glycopatterns between late-stage patients and healthy individuals, and between early-stage patients and patients with benign lung diseases.

A droplet microfluidics device was proposed to co-encapsulate EVs with gold nanoparticles coated by HER2-targeting aptamers, along with salt and Raman tags, for SERS detection^[46]^ - Figure 4B. The higher affinity between aptamers and HER2-positive EVs, together with the high-salt conditions, leads to nanoparticle aggregation and generation of strong SERS signals for identification of HER2^+^ EVs from breast tumor cells (LOD: 1 × 10^4.5^ particles/mL). A bimodal sensing approach, combining SERS with fluorescence, was also created^[47]^. The system used interdigitated transducers to generate acoustofluidic forces to control exosomes captured by CD63-aptamer-conjugated nanoparticles within a glass capillary. In fluorescent mode, fluorescently-labeled exosomes were concentrated at the center of the capillary for fluorescence quantification (LOD: 1.3 × 10^6^ particles/mL), while in SERS mode, by changing the acoustofluidic frequency, the EV-coated nanoparticles were re-focused to the wall of the capillary, previously coated with nanorods for enhanced SERS signaling, for EV detection from human plasma samples (LOD: 2 × 10^4^ particles/mL).

SERS-based methods offer highly sensitive detection and detailed molecular fingerprinting of EVs. However, they require complex plasmonic substrates and careful analysis to ensure batch-to-batch reproducibility of the nanostructures. This makes SERS ideal for applications requiring high precision and characterization, but less suitable for cost-effective, portable, and rapid point-of-care (PoC) testing. For applications where characterization, ultrasensitivity, and high specificity are critical, SERS is a powerful tool, whereas biosensors (such as electrochemical sensors) provide a more practical alternative when affordability, simplicity, and rapid detection are needed.

## SURFACE MARKER-INDEPENDENT EV QUANTIFICATION

Surface marker-independent EV quantification methods offer an alternative approach for detecting and analyzing EVs based on their physical and chemical properties. These techniques eliminate specific labeling protocols, helping to preserve the native state of EVs and reduce potential biases. They often provide additional information, such as size distribution and surface charge, valuable for functional analyses and biomarker discovery. However, these methods also present challenges, particularly in distinguishing EVs from other particles of similar sizes. A summary of the main features of surface marker-independent quantification platforms is provided in Table 2.

**Table 2:** Summary of surface marker-independent on-chip quantification techniques (nonspecific labeling & label-free), and techniques for internal cargo quantification

**Authors**	**Type**	**Principle**	**Performance parameters**	**Sample type**	**Sample pre-processing**
Paganini *et al*.^[26]^	Fluorescence	Hydrodynamic focusing + Fluorescence-based diffusion sizing	Size range: nanometers to hundreds of nanometers Range: 10^10^ to 10^15^ particles/mL Volume: 2-4 µL Completion time in minutes Differentiates EVs from co-isolated contaminants	CM	UC and UF
Friedrich *et al*.^[48]^	Fluorescence	Nanofluidic-based fluorescence flow cytometry	Size range: down to 100 nm Sensitivity: 170 fM to 500 pM vesicle concentration Time: < 2 min Volume: 20 μL sample volume	CM	UC
Hong *et al*.^[51]^	Fluorescence	Opto-thermo-electrohydrodynamic tweezer + fluorescence	Size: 30 and 150 nm Range: down to 1.8 × 10^5^ particles/mL	Commercial solution (EV standard)	Not applicable (EV standard)
Gustafson *et al*.^[49]^	Fluorescence	DEP collection + fluorescence quantification	Not provided	Plasma	N
Ware *et al*.^[50]^	Fluorescence	DEP collection + fluorescence quantification	LOD: 4.31 × 10^9^ BMVs/mL in plasma Linear range: 2.8 × 10^9^ to 7 × 10^9^ BMVs/mL Reproducibility: CV = 0.74 Volume: 25 µL	Plasma	N
Kim *et al*.^[52]^	Electrical	Resistive pulse sensing	High sensitivity (error of < 10%) Throughput: > 200,000 EVs/s Lower size limit: 50-100 nm	CM	UC
Young *et al*.^[53]^	Electrical	Resistive pulse sensing	60 to 160 nm Particle frequencies up to 1,000 per min Allows for measuring zeta potential	CM Milk	UC
Calado *et al*.^[54]^	Electrical	Resistive pulse sensing	Size: down to 80-100 nm Range: 10^6^-10^10^ particles/mL Throughput: 80 nL/s with particle concentrations up to 10^9^/mL	CM Human serum	UC
Cimorelli *et al*.^[55]^	Electrical	Resistive pulse sensing	Size: 65-75 nm up to 2 μm Range: 10^4^-10^12^ particles/mL	CM Urine Plasma	Minimal (centrifugation and collected supernatant + dilution)
Jalali *et al*.^[56]^	SERS	Nanostructured device with plasmonic nanobowties	LOD: 1.32 × 10⁵ particles/mL Differentiation between glioblastoma EVs, non-cancerous EVs, and synthetic liposomes	CM	Filtration and SEC
Cheng *et al*.^[60]^	Cargo	FET-based sensing	Sensitivity: miR-21: LOD of 6.07 fM/miR-126: LOD of 23.82 fM	CM Plasma	UC
Sung *et al*.^[59]^	Cargo	Digital PCR quantification	LOD: 11 copies/mL for miRNA-21 Accuracy: quantification accuracy of > 88% Time: EV isolation: 4 h; miRNA extraction: 20 min	Plasma	Magnetic immunoassay and lysis
Zhang *et al*.^[61]^	Cargo	PCR-free digital bioassay	LOD: 18 aM for GAPDH mRNA Specificity: discrimination between EWS-FLI1 fusion transcript variants Efficiency: GAPDH mRNA: 43.5-64.6 copies/10^5^ EVs. EWS-FLI1 mRNA: 0.277-6.5 copies/10^5^ EVs	CM	UC and lysis
Ramshani *et al*.^[58]^	Cargo	SAW-based lysing chip and IEM-based sensing	LOD: 1 pM for miR-21 Quantification accuracy: < 10% uncertainty for miRNA levels Specificity: differentiated between free-floating and EV-associated miRNAs	Plasma	N
Li *et al*.^[57]^	Cargo	Electrochemical sensing with a sandwich immunosensor	Sensitivity: 1 pg/mL for L1CAM Time: 1.5 h Sample Volume: 300 μL serum Specificity: high specificity demonstrated against interference proteins like IgG, CD81, HSA, and IL-6	Serum	N

BMVs: Bacterial membrane vesicles; CV: coefficient of variation; DEP: dielectrophoresis; EV: extracellular vesicle; FET: field-effect transistor; GAPDH: glyceraldehyde 3-phosphate dehydrogenase; HSA: human serum albumin; IL-6: interleukin 6; IEM: ion-exchange membrane; PCR: polymerase chain reaction; SAW: surface acoustic wave; SERS: surface-enhanced Raman scattering; LOD: limit of detection; CM: conditioned media; UC: ultracentrifugation; UF: ultrafiltration; SEC: size exclusion chromatography.

### Nonspecific labeling

**Figure 5:**
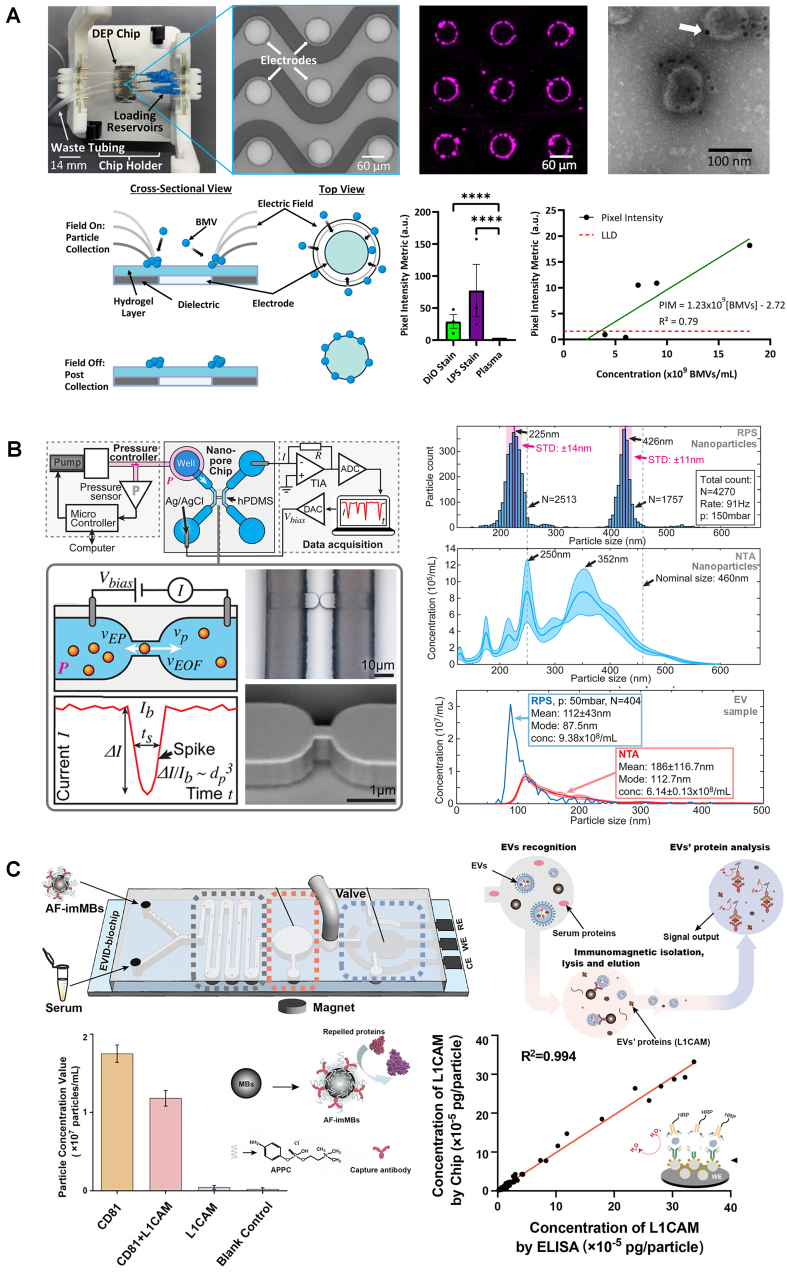
Surface marker-independent EV quantification and EV cargo quantification. (A) DEP-based EV capturing and quantification (adapted from ref.^[50]^); (B) Nanofluidic resistive pulse sensing of EVs (adapted from ref.^[54]^); (C) L1CAM quantification from L1CAM-positive EVs (adapted from ref.^[57]^). EVs: Extracellular vesicles; DEP: dielectrophoretic; BMV: bacterial membrane vesicles; RPS: resistive pulse sensing; NTA: nanoparticle tracking analysis.

These techniques use fluorescent nonspecific lipid dyes to stain the lipid bilayer of EVs for fluorescence-based quantification/analysis. This is the case of a nanofluidic flow cytometer for single lipid vesicles and EVs that enabled quantification and size distribution analysis as fluorescently labeled vesicles flow through parallel nanochannels under pressure-driven flow^[48]^. Single-particle analysis was performed by recording fluorescence signals, achieving detection of EVs down to 100 nm and quantification in the range of 170 fM to 500 pM. A different approach was a device that quantified fluorescent EVs while measuring the size distributions from their diffusion profiles^[26]^. This approach can detect EVs in a wide size range, in concentrations from 10^10^ to 10^13^ particles/mL, and differentiate EVs from co-isolated contaminants. Other groups have incorporated external fields into devices. The Ibsen group^[49,50]^ developed a label-free EV quantification technique in blood plasma by employing DEP for EV collection and automating fluorescence quantification. This approach was applied to quantify bacterial membrane vesicles (BMVs) in the range of 2.8-7 × 10^9^ BMVs/mL^[50]^ - Figure 5A. These techniques were only validated with fluorescent immunostaining, but, in principle, can be easily turned into label-free techniques using nonspecific labels. A similar approach used opto-thermo-electrohydrodynamic tweezers for trapping and manipulation of single exosomes (30-150 nm)^[51]^.

### Label-free

Label-free techniques eliminate specific labeling protocols by exploiting the physical or intrinsic chemical properties of EVs. This helps preserve their native state and reduces potential biases, often providing additional information like size distribution. However, the size and heterogeneity of EVs, and the presence of similar particles in samples, make label-free technique development challenging, requiring robust designs to ensure specificity.

Resistive pulse sensing (RPS) is a well-established EV quantification approach. Based on resistive signals generated when particles traverse a nanopore, RPS has been integrated into microfluidic devices in several configurations. A multi-gate design was proposed to enhance throughput, including a sheathless focusing detection gate with three nanochannels and a reference gate for noise reduction^[52]^. A different approach used a multi-pore nanochannel device that also enabled the determination of the zeta-potential of the EVs^[53]^. More recently, a RPS chip was developed with two parallel microchannels connected by in-plane nanopores^[54]^ - Figure 5B. This approach enabled calibration-free size distribution measurements across a dynamic range of 10^6^ to 10^10^ particles/mL, but provided only orders of magnitude estimates for the concentration. Microfluidic RPS has even been commercialized, as exemplified by Spectradyne LLC (Torrance, CA, USA), which offers a system with microfluidic cartridges containing a nanoconstriction. This platform has been used to optimize a standardized procedure for measuring EV concentration and size distribution in unpurified biofluid samples from various sources^[55]^. While RPS systems excel in scalability, cost-effectiveness, and adaptability for high-throughput applications, their performance is constrained by the nanopore size. Furthermore, these devices are susceptible to clogging and often suffer from reproducibility limitations. The fabrication of nanopores also presents challenges, being complex, time-consuming, and costly.

An additional strategy was to use SERS by embedding the nanostructures in the microchannels. For instance, plasmonic nanobowties were used to amplify EV Raman signals and detect and analyze EVs in the samples, achieving a LOD of 1.32 × 10^5^ particles/mL^[56]^.

## EV INTERNAL CARGO QUANTIFICATION

Microfluidic systems streamline the quantification of EV cargo biomarkers by integrating on-chip isolation, lysis, and analysis. One notable approach was a microfluidic electrochemical sandwich immunosensor [Figure 5C] for detecting L1CAM, a proposed biomarker for Parkinson’s disease. The system used immunomagnetic beads for EV isolation, on-chip lysis, and electrochemical protein detection, achieving a sensitivity of 1 pg/mL for L1CAM, with high specificity against potential interference proteins such as IgG, CD81, HSA, and IL-6^[57]^.

MicroRNAs (miRNAs) are another class of biomarkers commonly analyzed. For example, a microfluidic platform incorporating surface acoustic waves for EV lysis and ion-exchange membranes for miRNA concentration and detection was developed^[58]^. This system quantified miR-21 with a detection limit of 1 pM and distinguished between free-floating and EV-associated miRNAs, enabling precise analysis of plasma samples. The Gwo-Bin Lee group^[59,60]^ advanced automated microfluidic systems for miRNA quantification by combining CD63 antibody-coated magnetic beads for EV isolation with probe-coated magnetic beads for miRNA extraction from EV lysates. Using these systems, the group developed two quantification modules. A digital PCR module enabled miR-21 quantification with a detection limit of 11 copies/mL in ovarian cancer samples. A second approach used sensors based on field-effect transistors, achieving detection limits of 6.07 fM for miR-21 and 23.82 fM for miR-126, highlighting their versatility in detecting disease-related miRNAs. For mRNA analysis, a microwell-patterned microfluidic device for highly sensitive digital bioassays enabled absolute quantification of mRNA biomarkers from EV lysates using dual-probe hybridization and enzymatic signal amplification^[61]^. Using dual-probe hybridization and enzymatic signal amplification, the platform enabled absolute quantification of mRNA biomarkers from EV lysates. However, unlike other platforms, this system requires off-chip EV isolation and lysis prior to on-chip analysis.

## DISCUSSION AND FUTURE PERSPECTIVES

EVs hold significant promise as biomarkers for diagnosis, prognosis, and treatment monitoring, as well as in drug delivery systems. Accurate quantification and characterization are therefore crucial to realizing their potential in these applications. However, current techniques for EV analysis are often costly and require specialized equipment and skilled operators. Microfluidic technology offers a potentially more accessible alternative. While most of the on-chip platforms focused primarily on EV capture and isolation, recent scientific exploration has increasingly focused on the development of microfluidic technologies for EV quantification and characterization. In this work, we have reviewed the most recent advances in this field. Most of the systems discussed are based on the presence of surface markers, either those commonly found on EVs (e.g., CD9, CD63, CD81), or disease-specific proteins of interest (e.g., HER2, CD24, EpCAM, PD-1, PD-L1). The measurement of the fluorescent intensity when EVs were captured was the most common approach. Electrochemical and SERS-based systems have also shown promise for detailed EV subpopulation studies relevant to clinical diagnosis. Some of these works employed droplet microfluidics^[24,29,46]^, a branch of microfluidics dealing with segmented flow that is very well known for its application in the analysis of single cells. Researchers in both fields have adapted those technologies to EV analysis, enabling subpopulation studies of EVs expressing specific target proteins, which may contribute to unlocking the potential of EVs in clinical diagnosis. Droplet microfluidics offers significant advantages for EV analysis, including high-throughput capabilities and precise microenvironment control. For instance, simultaneous detection of membrane proteins and mRNA at the single EV level was achieved with high fidelity^[62,63]^. Similarly, Tong *et al*.^[64]^ developed an all-in-one microfluidic workstation for high-throughput EV miRNA detection in non-small cell lung cancer. Further, Reynolds *et al*.^[65]^ presented a double digital assay for single EV and single-molecule detection, again showing the potential for high-sensitivity analysis of individual EVs and their molecular cargo using microdroplet-enabled strategies. In addition, nonspecific labeling and label-free methods enable the quantification of intact EVs, suitable for subsequent functional studies or heterogeneous EV populations. Quantification of EV internal cargo, such as proteins and nucleic acids, has also been explored.

Comparing these strategies reveals that each has unique strengths and limitations, and that the choice between these approaches often depends on the intended application. Surface marker-independent approaches offer the advantage of preserving the native state of EVs, minimizing labeling biases, and enabling simultaneous analysis of physical properties. RPS integrated into high-throughput microfluidic devices offers scalability and cost-effectiveness. However, distinguishing EVs from similarly sized particles in complex samples and addressing EV population heterogeneity remain challenges. In contrast, surface marker-specific quantification techniques leverage EV membrane biomarkers for detection and quantification. Fluorescence-based assays, for example, often utilize fluorophore-conjugated antibodies to selectively bind to surface markers, enabling high sensitivity and even the capability to perform single-particle analysis. However, specific labeling may sometimes alter the native state of the EVs or introduce variability based on antibody performance. Colorimetric assays offer similar specificity and easier adaptation to PoC settings but may lack the quantitative precision and dynamic range of other methods. Electrochemical methods provide high sensitivity and rapid response times, are cost-efficient, and are well-suited for integration into portable platforms, yet their performance depends on antibody quality and immobilization characteristics. SERS-based quantification techniques, while sometimes grouped with surface marker-independent methods due to their reliance on plasmonic nanostructures, can be adapted for surface marker-specific detection. By functionalizing plasmonic substrates with specific antibodies, SERS enables both molecular fingerprinting and targeted EV recognition, achieving low detection limits and detailed biochemical information on EV cargo. However, the need for precisely engineered nanostructures and complex spectral interpretation often restricts SERS to specialized research rather than routine clinical diagnostics^[66]^.

Microfluidic technologies offer several advantages over other analytical techniques, including minimal sample handling (i.e., no EV pre-isolation), short processing times (5-90 min), small starting volumes (hundreds of µL of biofluid), and the ability to purify less abundant EV populations. On-chip EV quantification could represent a suitable alternative to NTA, with the added benefit of data on EV sub-populations and phenotypes. While some NTA devices have a limited LOD of ~10^5^-10^6^ particles/mL, several microfluidic techniques demonstrate significantly lower LODs. Furthermore, microfluidic platforms can integrate sample pre-processing steps (e.g., isolation, concentration, labeling), enabling online EV quantification from whole samples. However, challenges remain in the mass-scale production of microfluidic devices, impacting reproducibility, robustness, and interuser/interlab variability. Although techniques like hot-embossing and injection molding are used for large-volume production, they are primarily suited for devices with medium or large features (above 20 µm)^[67]^. Smaller features limit the resolution and fidelity of mold replicas. Advances in 3D printing and roll-to-roll production are expanding the scalability of these devices, which will enhance data-backed validation of microfluidic platforms for EV quantification. Increased automation and integration with high-throughput systems would further improve reproducibility and scalability. Standardization of protocols, validation against conventional techniques, and compliance with regulatory frameworks [e.g., Food and Drug Administration (FDA) or Conformité Européenne (European Conformity, CE) marking requirements] are crucial for the widespread adoption of microfluidic devices in research and clinical applications. Additionally, the microfluidic platforms discussed in this review have been predominantly applied to serum, plasma, or whole blood. Thus, exploring their application for quantifying EVs from other bodily fluids holds potential for biomedical research, although regulatory and standardization hurdles must be addressed. It is also worth highlighting that, beyond the use of NTA as benchmarking for the development and validation of on-chip quantification, the combination of both techniques may be beneficial. Using NTA for quality sample testing, mostly when designing protocols with cell media and model cell lines, followed by on-chip quantification, may advance the field by facilitating the exploration and understanding of EVs and their clinical validation. Lastly, in light of the above-mentioned limitations, the community of EV researchers would benefit from incorporating the data generated in these studies into platforms such as EV-TRACK^[68]^, which are specifically designed to centralize and share comprehensive data from EV-related studies. This platform includes information on the isolation and characterization techniques of EVs, as well as their origin (e.g., species or type of biofluid). For microfluidic quantification technologies, a broad classification could be established based on the main approaches suggested in this review: surface-based or surface marker-independent (nonspecific or label-free). The selection of the strategy depends on the intended application: the study of biomarkers located on the surface or contained within the internal cargo of EVs, functional studies, quantification and characterization, *etc*. Performance parameters, such as the LOD, capture efficiency, linear range, or reproducibility, are reporting elements that provide valuable information for researchers who may be interested in using microfluidics for their studies. Additionally, specific data on these devices could be included, such as an open repository of the microfluidic designs, fabrication methods, flow rates used, volumes sustained, operational mode, materials, or functionalization protocols. Among the most relevant aspects are the surface and/or nanoparticle functionalization methods used, whether they operate in continuous flow or perform measurements after sample processing, and whether they integrate both separation and quantification or focus solely on quantification. Addressing these challenges and incorporating growing knowledge in the EV field will facilitate the clinical and other applications of microfluidic EV analysis tools.

## DECLARATIONS

### Copyright

© The Author(s) 2025.
